# Ectromelia-encoded virulence factor C15 specifically inhibits antigen presentation to CD4^+^ T cells post peptide loading

**DOI:** 10.1371/journal.ppat.1008685

**Published:** 2020-08-03

**Authors:** Katherine S. Forsyth, Nathan H. Roy, Elise Peauroi, Brian C. DeHaven, Erik D. Wold, Adam R. Hersperger, Janis K. Burkhardt, Laurence C. Eisenlohr

**Affiliations:** 1 Department of Pathology and Laboratory Medicine, Perelman School of Medicine, University of Pennsylvania, Philadelphia, Pennsylvania, United States of America; 2 Department of Biology, La Salle University, Philadelphia, Pennsylvania, United States of America; 3 Department of Biology, Albright College, Reading, Pennsylvania, United States of America; 4 Children's Hospital of Philadelphia Research Institute, Philadelphia, Pennsylvania, United States of America; University Medical Center Utrecht, NETHERLANDS

## Abstract

Smallpox and monkeypox pose severe threats to human health. Other orthopoxviruses are comparably virulent in their natural hosts, including ectromelia, the cause of mousepox. Disease severity is linked to an array of immunomodulatory proteins including the B22 family, which has homologs in all pathogenic orthopoxviruses but not attenuated vaccine strains. We demonstrate that the ectromelia B22 member, C15, is necessary and sufficient for selective inhibition of CD4^+^ but not CD8^+^ T cell activation by immunogenic peptide and superantigen. Inhibition is achieved not by down-regulation of surface MHC- II or co-stimulatory protein surface expression but rather by interference with antigen presentation. The appreciable outcome is interference with CD4^+^ T cell synapse formation as determined by imaging studies and lipid raft disruption. Consequently, CD4^+^ T cell activating stimulus shifts to uninfected antigen-presenting cells that have received antigen from infected cells. This work provides insight into the immunomodulatory strategies of orthopoxviruses by elucidating a mechanism for specific targeting of CD4^+^ T cell activation, reflecting the importance of this cell type in control of the virus.

## Author summary

Orthopoxviruses pose considerable threats to their hosts by producing a battery of proteins that disable the immune system at many levels through mechanisms that remain poorly understood. An essential part of most immune responses is the activation of CD4^+^ T cells by antigen-presenting cells through formation of a supramolecular structure termed the immunological synapse. We show here that the C15 protein of ectromelia, the cause of mousepox, inhibits CD4^+^ T cell activation through a novel immunoevasion mechanism that results in disruption of synapse formation. As many poxviruses encode C15 homologs, these studies could provide insights into the virulence of other family members including monkeypox and smallpox, both of great concern to human populations.

## Introduction

CD4^+^ T cells are a critical adaptive immune cell type with roles in B cell and CD8^+^ T cell help, inflammatory cytokine secretion and, in some cases, direct cytolytic function. The critical initiating events for CD4^+^ T cell activation are Major Histocompatibility Complex class II (MHCII) presentation of pathogen derived peptides (epitopes) in combination with co-stimulatory signals through CD28 [1]. As CD4^+^ T cells play important roles in clearing many viral infections, several mechanisms by which viruses inhibit MHCII antigen processing and presentation have been described [2]. For example, most steps of the MHCII maturation process are targeted, from inhibition of the master transcription factor CIITA [3–8] to interference with complex formation and trafficking [9–11], as well as forced degradation of mature MHCII molecules [12, 13]. In addition, Hepatitis C Virus inhibits the function of endosomal proteases required for generating some MHCII binding peptides [14]. Furthermore, Epstein-Barr virus (EBV) utilizes a soluble factor to block MHCII engagement with the T cell receptor via steric hindrance [15, 16]. Of note, though discrete mechanisms of inhibition have been described, there are relatively few examples of CD4^+^ T cell inhibition in the literature compared to the many of viral inhibition of MHCI presentation to CD8^+^ T cells, perhaps reflecting historical inattention to the role of CD4^+^ T cells in viral clearance.

Another possible explanation for the dearth of inquiries into viral targeting of CD4^+^ T cells is the prevailing view of the requirements for MHCII processing of antigen. The classical view of MHCII presentation begins with internalization of extracellular material by a professional antigen-presenting cell (APC), followed by processing of antigen within the endocytic network and peptide loading onto nascent MHCII molecules in the late endosomal compartment [1]. However, the presentation of endogenously produced antigen following infection of the APC via non-canonical processing pathways has been described in many viral systems [17–24]. Indeed, we have recently reported that during influenza infection the majority of MHCII presentation derives from endogenously produced proteins by directly infected APCs [24]. Importantly, the efficacy of inhibitory mechanisms that target MHCII processing and presentation generally necessitate direct infection of the APC. This reinforces the importance of direct presentation by the infected APC in host defense.

Many orthopoxviruses can cause severe disease in humans, most notably smallpox, one of the deadliest diseases in human history and an ongoing bioterrorism threat [25, 26]. Furthermore, zoonotic orthopoxviruses have emerged as novel disease causing agents; monkeypox (MPXV) is passed human-to-human and is evolving to target the human immune system more effectively [27]. Camelpox, cowpox and buffalopox can also cause severe infections in humans [27]. Even vaccinia (VACV), the gold standard smallpox vaccination, is itself a live virus that can cause disease and secondary transmission with potentially severe clinical complications in contraindicated persons [28].

Orthopoxviruses are the largest of the mammalian DNA-based viruses, encoding approximately 200 open reading frames (ORFs). Orthopoxviruses have strong conservation of viral replication proteins but contain more diversity in the regions of the genome implicated in virulence [29–31]. For example, virulence factors interfere with interferon signals, block cytokine signaling and impede innate intracellular antiviral pathways [32–51]. Importantly, many of these ORFs have not yet been ascribed a specific function. Thus, many interactions between orthopoxvirus proteins and host factors have not yet been investigated. To this end, we utilized the orthopoxvirus ECTV, as it is the causative agent of mousepox and has evolved as a natural mouse pathogen [52]. Importantly, ECTV displays a similar disease profile as smallpox or MPXV [52, 53]. In addition, ECTV causes much greater pathology in the mouse than the smallpox vaccine virus vaccinia (VACV), despite their high degree of homology (>93%), highlighting the importance of analyzing virulence factors in a setting that provides their natural targets.

CD4^+^ T cells are critical for the clearance of ECTV, helping to produce a strong neutralizing antibody response as well as displaying cytolytic function in directly killing infected cells [54, 55]. Therefore, a strategy to target CD4^+^ T cells would be consistent with the major investment in immune evasion characteristic of all orthopoxviruses. Here, we demonstrate that C15, the largest immune evasion protein by size in the ECTV arsenal, a member of the B22 family of proteins, and a considerable virulence factor [56], is necessary and sufficient to inhibit CD4^+^ but not CD8^+^ T cell activation via interference with synapse formation.

## Results

### Direct endogenous MHCII presentation is inhibited by ECTV

**Fig 1:**
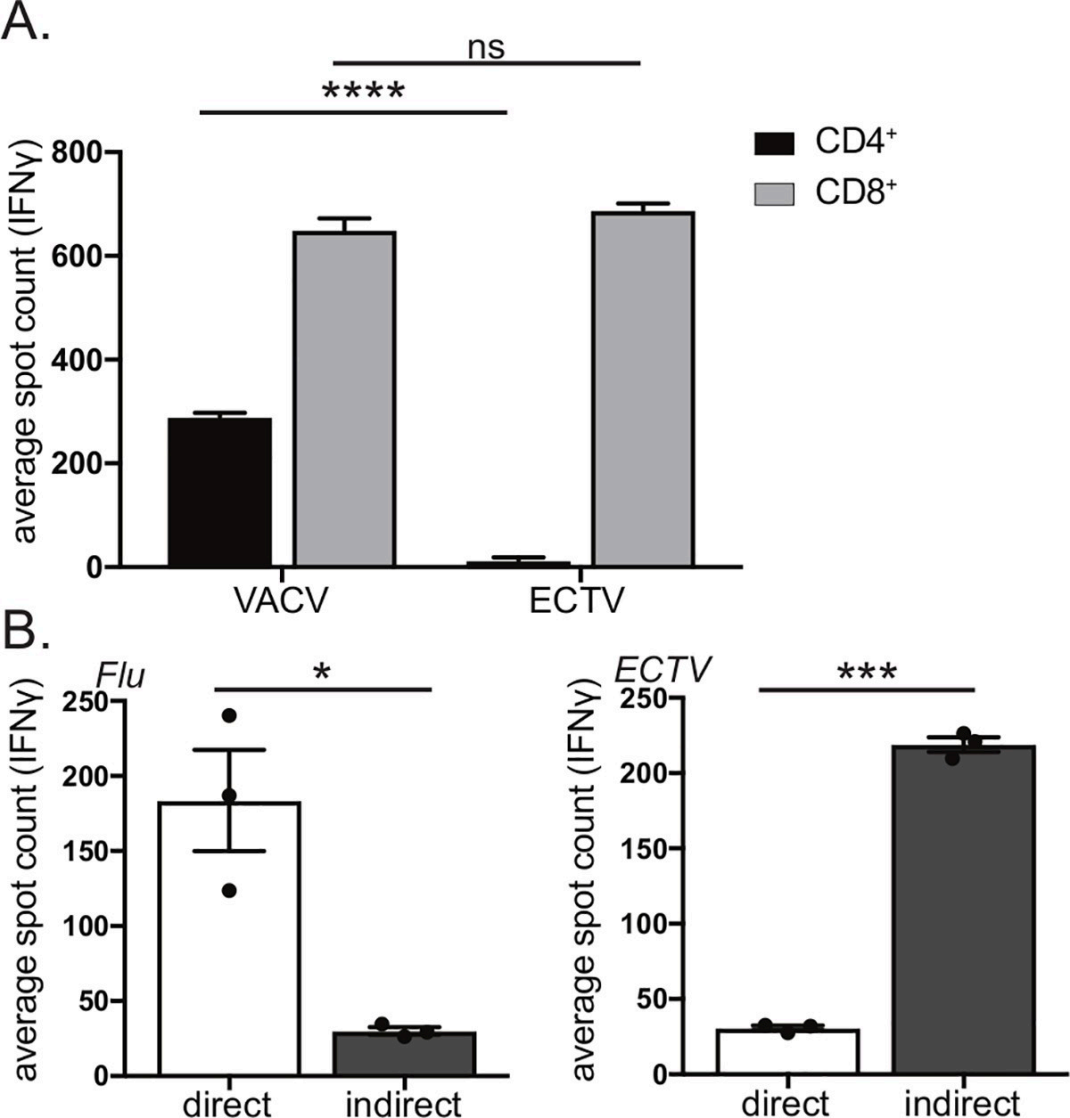
ECTV blocks direct MHCII presentation to CD4^+^ T cells. **A)** *ECTV specifically targets MHCII but not* MHCI *presentation*. ECTV-specific CD4^+^ and CD8^+^ T cells were co-incubated with BMDCs infected with either VACV or WT ECTV and analyzed for IFNγ production by ELISpot. **B)** *Bias of influenza to direct MHCII presentation in contrast to ECTV*. PR8 influenza-specific or ECTV-specific CD4^+^ T cells were co-incubated in the presence of neutralizing antibody with either BMDCs infected with virus (direct presentation) or infected fibroblasts and uninfected BMDCs (indirect presentation). CD4^+^ T cell activation was measured via IFNγ production by ELISpot. Each dot represents the mean spot count of technical replicates of an independent infection of BMDCs or fibroblasts, with three infections per condition. Representative of three-eight independent experiments. Significance analyzed by student’s T test, * p<0.05, ***p<0.001, **** p<0.0001, error bars signify (A) square root of squared SEMs or (B) SEM.

As ECTV is considerably more pathogenic in mice than the vaccine virus VACV, we initially asked whether T cells, either CD4^+^ or CD8^+^, would respond differently to ECTV infection compared to VACV infection. We isolated CD4^+^ and CD8^+^ T cells from the spleens of ECTV-infected mice and co-cultured with bone marrow derived dendritic cells (BMDCs) infected with either ECTV or VACV. T cell activation was measured using an IFNγ ELISpot. While we observed robust CD8^+^ T cell activation to BMDCs infected with either virus, CD4^+^ T cell activation occurred only in the VACV-infected condition with minimal response to ECTV-infected BMDCs (**Fig 1A**). This result suggests selective targeting of CD4^+^ T cell activation by ECTV.

We have previously reported on robust CD4^+^ T cell responses following ECTV infection [57–59]. In light of this, the observed inhibitory effect suggests that MHCII processing and presentation of ECTV antigens relies on indirect presentation of antigen by an uninfected APC rather than direct presentation. As we have previously shown that direct endogenous presentation is the major driver of MHCII presentation during influenza infection [24], we compared modes of MHCII antigen presentation during influenza and ECTV infection. We isolated CD4^+^ T cells from the spleens of influenza or ECTV infected mice, co-cultured with BMDCs presenting MHCII peptides derived from either direct presentation or indirect presentation and measured T cell activation using an IFNγ ELISpot (**Fig 1B**). The direct presentation condition utilized virus-infected BMDCs, while the indirect presentation condition employed virally infected MHCII^-^ fibroblasts that would require antigen to be transferred to BMDCs to allow for CD4^+^ T cell activation. Neutralizing antibody was included to prevent the spread of infectious virus to BMDCs, thereby blocking the possibility of direct presentation. Replicating our previously published findings [24], we determined that for influenza virus, direct presentation resulted in significantly more robust CD4^+^ T cell activation compared to indirect presentation (**Fig 1B**). For ECTV, in contrast, indirect presentation resulted in much stronger CD4^+^ T cell activation compared to direct presentation (**Fig 1B**). Thus, ECTV-mediated inhibition of CD4^+^ T cell activation can be bypassed via indirect presentation of antigen transferred to uninfected APCs.

### C15 is an ECTV virulence factor but does not impact viral replication

**Fig 2:**
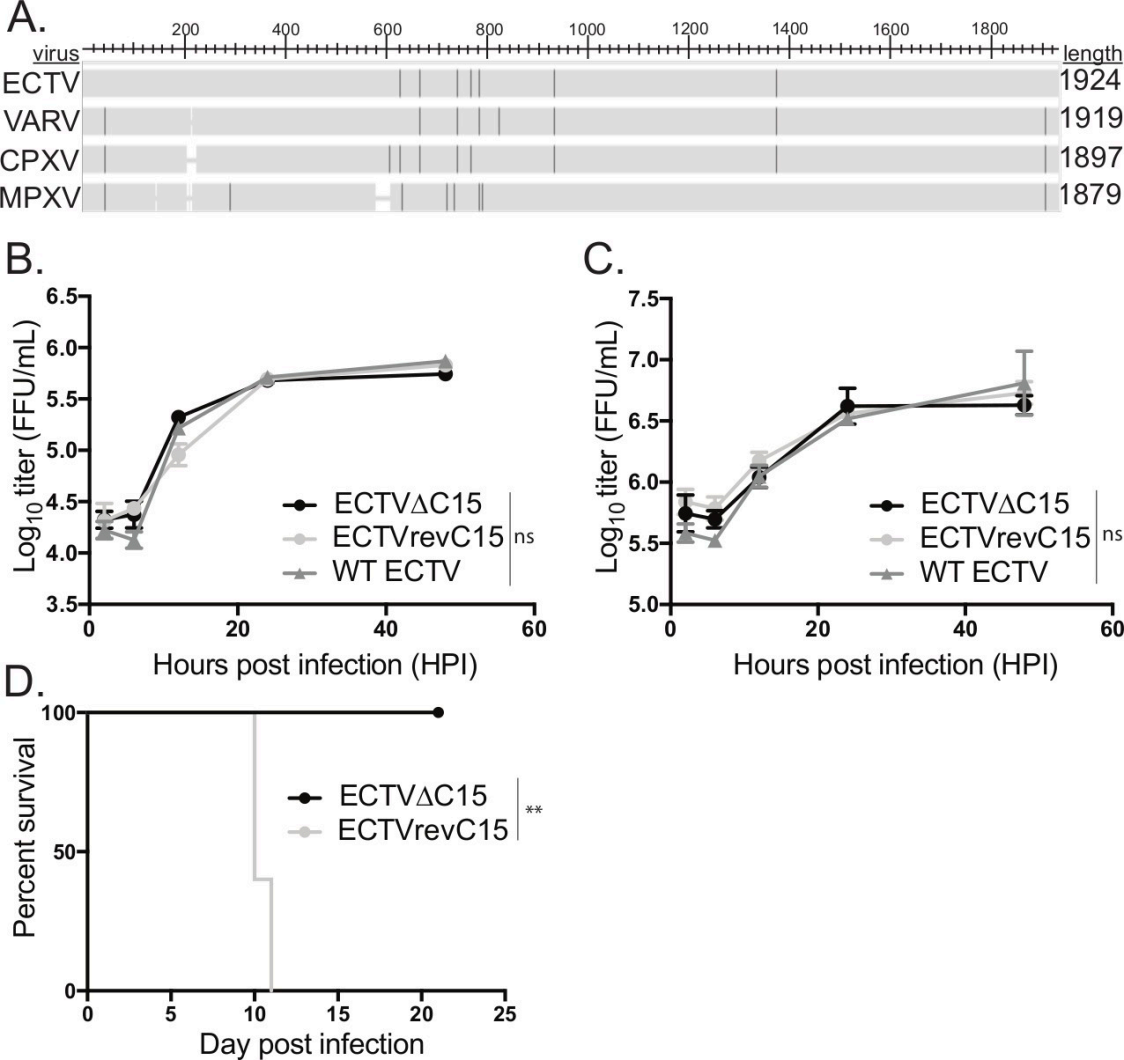
C15 is a virulence factor that is not required for viral replication. **A)** Amino acid sequences for the B22 family members in ECTV Moscow, VARV BG1975, CPXV Brighton Red or MPXV Zaire were analyzed for sequence similarity via pBLAST (NCBI), with sequence differences highlighted. Sequences were obtained from the Viral Bioinformatics Resource Centre. **B-C)** Either **B)** C57Bl/6 fibroblasts or **C)** BMDCs were infected with WT ECTV (GFP), ECTVΔC15 or ECTVrevC15 at a MOI of 3 in triplicate and infectious lysates were harvested at various times post infection and analyzed for infectious units via focus forming assay. Representative of two independent experiments. Significance analyzed by two way ANOVA, error bars signify SEM. **D)** BALB/c mice were infected with either ΔC15 ECTV or ECTVrevC15 and morbidity and mortality were assessed daily. Representative of two independent experiments. Significance analyzed by Kaplan-Meier estimate, **p<0.01.

To identify the factor responsible for CD4^+^ T cell inhibition by ECTV, we turned to the B22 family of proteins, named for the B22 open reading frame (ORF) in smallpox. A B22 family protein is present in all pathogenic orthopoxviruses, including ECTV, but is highly truncated in VACV (~1% of the coding sequence remains) (**Fig 2A**). In addition, previous reporting on the B22 family member of ECTV, C15, and B22 family members of other pathogenic poxviruses described general inhibition of T cell responses [56, 60]. To analyze whether the B22 protein encoded by ECTV, C15, played a role in the inhibitory phenotype observed above, we first generated a virus via standard recombination methods [61] in which the majority of the C15 coding sequence was replaced with the GFP gene (ECTVΔC15). Subsequently, we created a C15 revertant ECTV (ECTVrevC15) by homologous recombination.

To validate that C15 does not play a role in viral replication, we performed an *in vitro* growth curve in fibroblasts comparing ECTVΔC15, ECTVrevC15, and w.t. ECTV engineered to express GFP [62]. We observed that the loss of C15 has no effect on viral replication *in vitro*, which is often the case for immunomodulatory gene products (**Fig 2B**). In addition, we analyzed the *in vitro* replication kinetics in BMDCs and observed no impact of C15 on viral replication (**Fig 2C**). To analyze the impact of C15 on virulence, we infected ECTV-susceptible BALB/c mice with ECTVΔC15 or ECTVrevC15 and observed that C15 is a strong virulence factor *in vivo* since all mice infected with ECTVΔC15 survived (**Fig 2D**). Interestingly, footpad inflammation leading to foot necrosis was still observed in the mice infected with ECTVΔC15 **(S1 Fig).** Together these data are in agreement with the previous report that C15 is a virulence factor *in vivo* but is dispensable for viral replication [56].

### C15 restricts CD4^+^ but not CD8^+^ T cell activation

**Fig 3:**
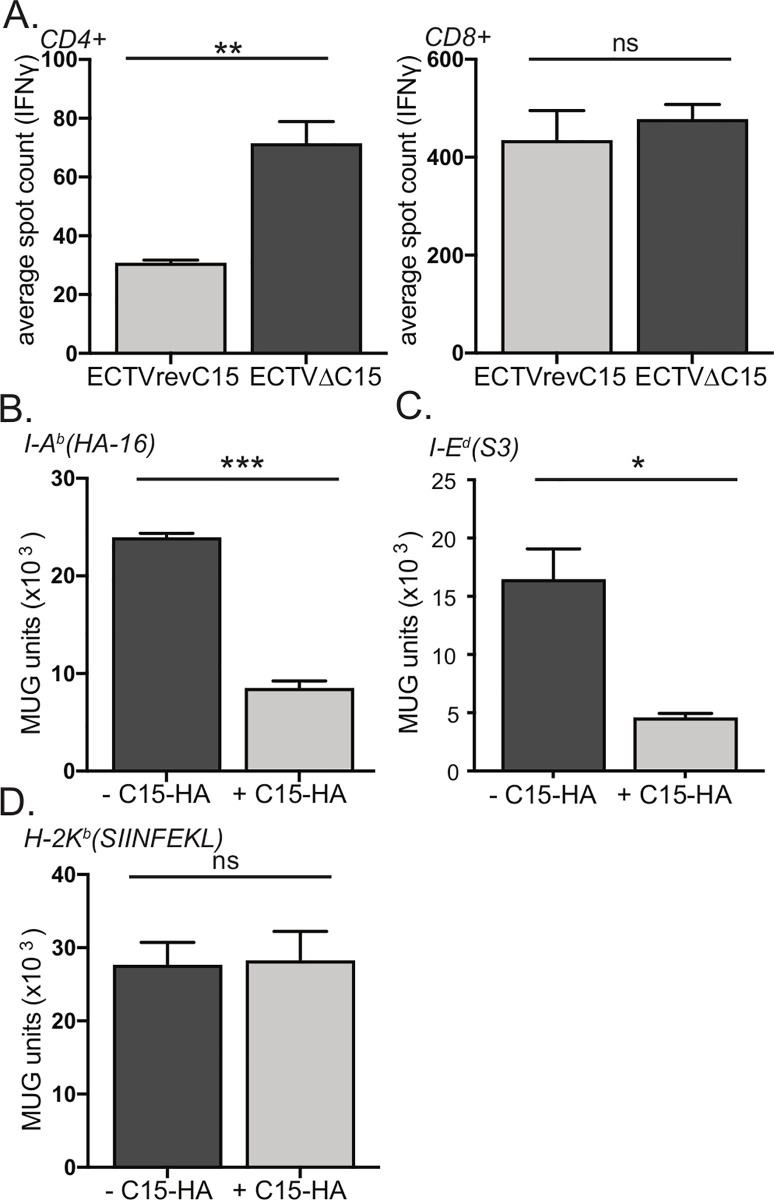
C15 is necessary and sufficient for selective inhibition of CD4^+^ T cell activation. **A)** ECTV-specific CD4^+^ or CD8^+^ T cells were co-incubated with BMDCs infected with ECTVΔC15 or ECTVrevC15 and analyzed for IFNγ production by ELISpot. Representative of three-eight independent experiments. **B-D)** B6-IE^d^ fibroblasts were transfected with C15-HA prior to staining for surface expression of HA tag and sorting based on HA tag expression. Cells were pulsed with **B)** HA-16 (I-A^b^ restricted), **C)** S3 (I-E^d^ restricted) or **D)** SIINFEKL (K^b^ restricted) peptide and then co-cultured overnight with T cell hybridomas specific for each peptide. T cell activation was measured by proxy of β-galactosidase conversion of MUG substrate. Representative of three independent experiments. Significance analyzed by student’s T test, * p<0.05, **p<0.01, *** p<0.001, error bars signify square root of squared SEMs.

Our data in Fig 1 suggest that ECTV specifically inhibits CD4^+^ T cell activation. However, published work with other B22 family members demonstrated a role for inhibition of both CD4^+^ and CD8^+^ T cells [60]. We therefore examined the role of C15 in blocking CD4^+^ and CD8^+^ T cell activation by isolating T cells, either CD4^+^ or CD8^+^, from the spleens of ECTV infected mice, co-culturing with BMDCs infected with either ECTVΔC15 or ECTVrevC15, and analyzing T cell activation via IFNγ ELISpot (**Fig 3A**). We observed that CD8^+^ T cells were robustly re-activated by the re-stimulating virus independent of C15 expression, but that CD4^+^ T cell responses were significantly suppressed in the presence of C15 (**Fig 3A**). In addition, we analyzed the relative contributions of direct and indirect presentation in the presence and absence of C15. In agreement with our hypothesis, we observed that C15 had no role in inhibiting indirect presentation but rather specifically inhibited direct presentation (**S2 Fig**). These data demonstrate that ECTV C15 is necessary to inhibit CD4^+^ but not CD8^+^ T cell activation. Moreover, C15 specifically targets direct presentation by MHCII, a fundamentally different mechanism of action relative to the MPXV homolog of B22.

### C15 is sufficient for selective inhibition of CD4^+^ T cell activation

In order to analyze the impact of C15 in isolation on T cell activation, we created a mammalian expression vector encoding C15 with a C-terminal HA tag (C15-HA). As has been previously shown for the VARV, MPXV, and CPXV homologs [60, 63] and predicted *in silico* [64], we observed the C terminus to be extracellular via flow cytometric analysis of the HA tag (**S3 Fig**). As transfection of BMDCs is technically challenging, we employed the C57Bl/6-CIITA-IE^d^ fibroblast cell line (B6-IE^d^), a tool we have used frequently to study MHCII presentation by both IA^b^ and IE^d^ MHCII molecules [22, 24, 65]. We transfected B6-IE^d^ cells with the C15-HA construct and sorted the populations based on HA-tag expression. We then pulsed both populations with specific IA^b^ or IE^d^- restricted influenza peptides and co-cultured APCs overnight with a panel of T cell hybridomas, each specific for a given peptide. For CD4^+^ T cell hybridomas specific for an IA^b^- restricted epitope (**Fig 3C**) or an IE^d^-restricted epitope (**Fig 3D**), the presence of C15 significantly impacted activation. In contrast, activation of a CD8^+^ T cell hybridoma was not diminished by the presence of C15 (**Fig 3E**). These data show that C15 is sufficient for selective inhibition of CD4^+^ T cell activation.

### C15 inhibition is independent of MHCII or co-activation protein surface downregulation

**Fig 4:**
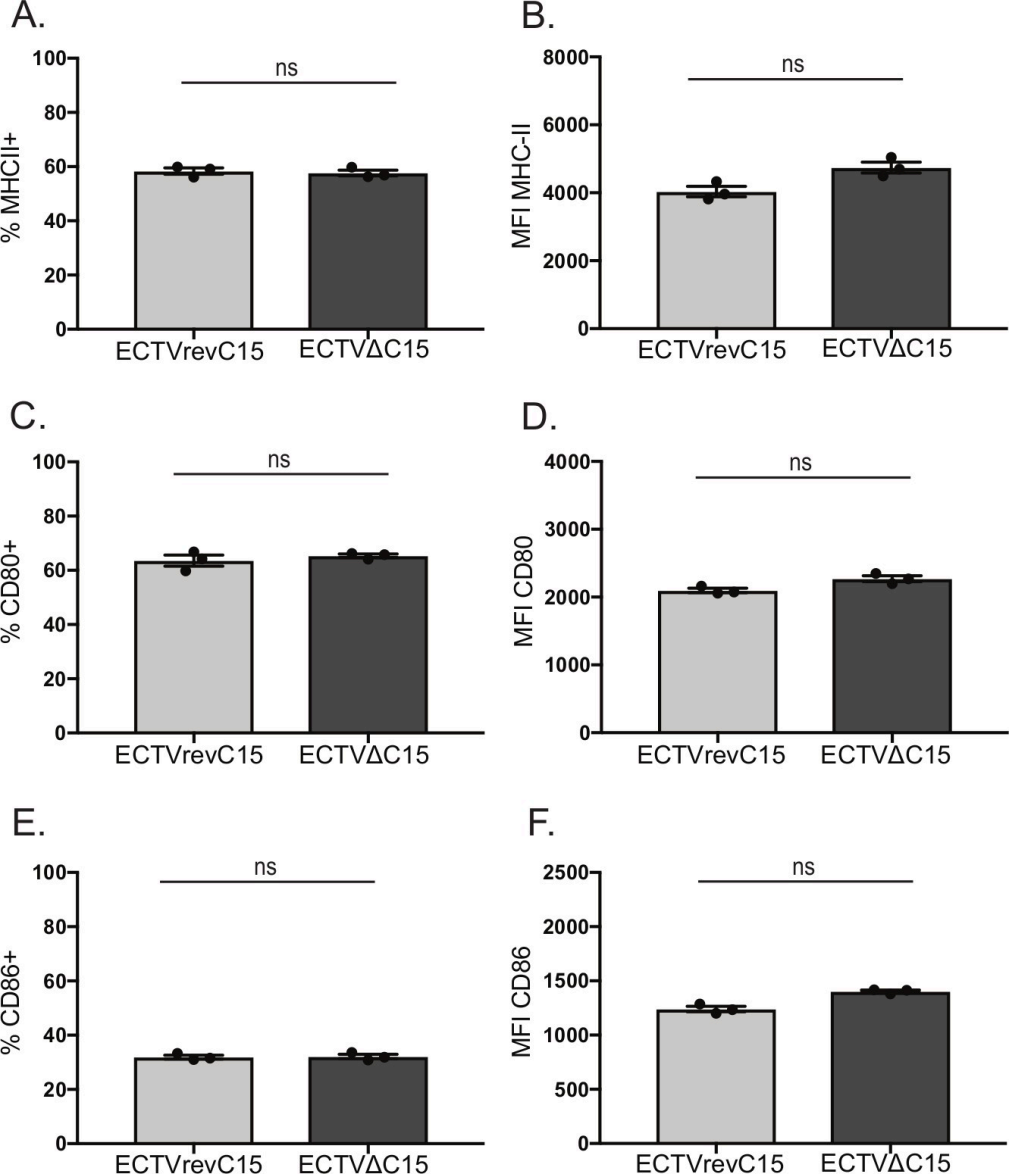
C15 does not inhibit surface expression of MHCII or co-activation proteins. BMDCs were infected independently with either ECTVΔC15 or ECTVrevC15 overnight and stained for surface expression of **A-B)** MHCII, **C-D)** CD80 or **E-F)** CD86. Flow cytometry data was analyzed for **A,C,E)** percent of cells expressing or **B,D,F)** mean fluorescence intensity for each protein. Each dot represents an independent infection of BMDCs, with three infections per condition. Representative of three independent experiments. Significance analyzed by student’s T test, error bars signify SEM.

Many viral proteins that inhibit MHCII processing and presentation act via downregulation of surface MHCII levels, either by blocking transcription or by degrading mature MHCII molecules [3–8, 12, 13]. To determine if C15 inhibits CD4^+^ T cell activation in this manner or by targeting the classical co-activation proteins CD80 and CD86, we infected BMDCs with either ECTVΔC15 or ECTVrevC15 overnight and analyzed surface levels of MHCII (**Fig 4A and 4B**), CD80 (**Fig 4C and 4D**) and CD86 (**Fig 4E and 4F**) via flow cytometry. We did not observe any differences in either the percent of cells expressing these proteins (**Fig 4A, 4C and 4E**) or levels expressed at the cell surface (mean fluorescence intensity, MFI) (**Fig 4B, 4D and 4F**). Therefore, C15 does not inhibit CD4^+^ T cell activation by downregulating surface levels of MHCII, CD80 or CD86.

### C15 inhibition of MHCII presentation is mediated *in cis*

**Fig 5:**
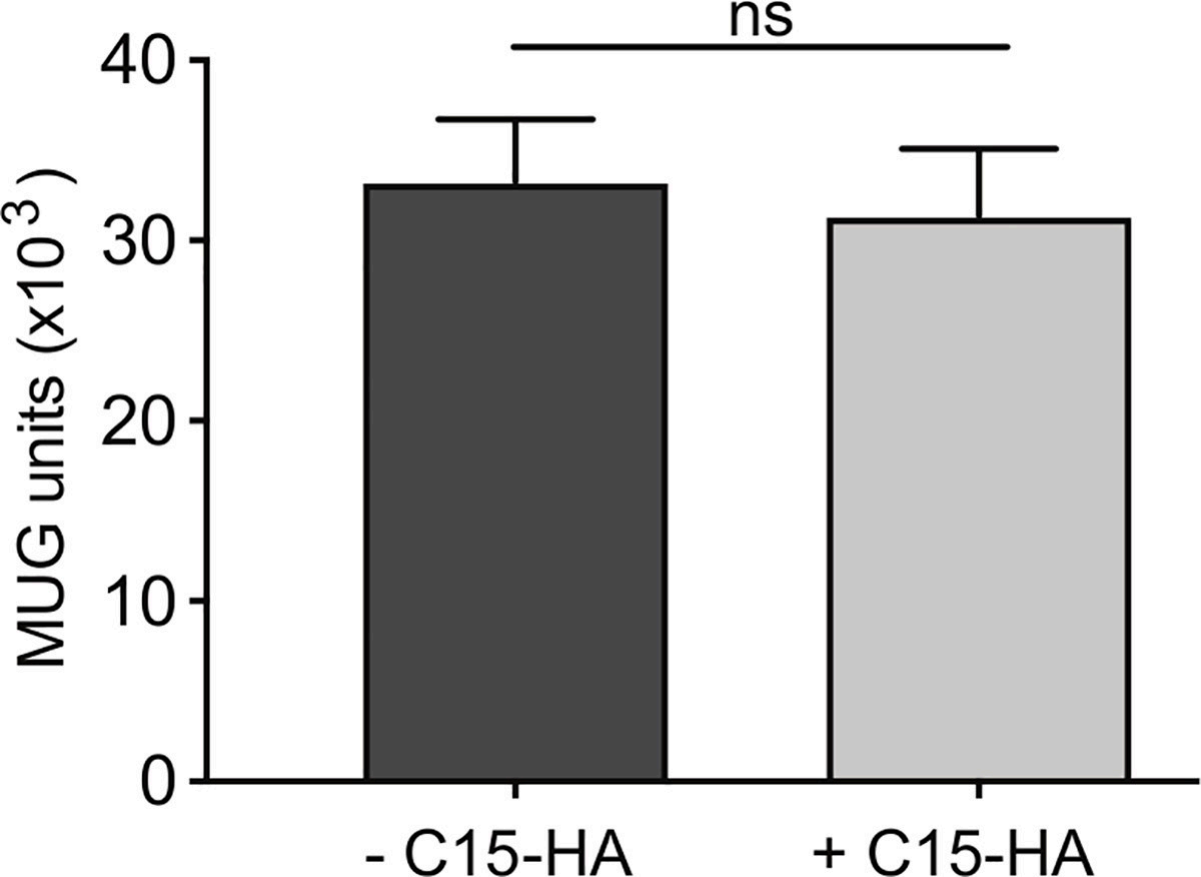
C15 inhibits MHCII presentation *in cis*. CHO cells were transfected with C15-HA for 24 hours. Cells were stained for surface expression of HA, sorted based on HA expression and co-cultured with BMDCs pulsed with HA-16 peptide and an HA-16 specific T cell hybridoma. T cell activation was measured by proxy of β-galactosidase conversion of MUG substrate. Representative of three independent experiments. Significance analyzed by student’s T test, error bars signify square root of the squared SEMs.

Since C15 does not function in a straightforward manner by downregulation of MHCII or co-stimulatory proteins, we analyzed whether C15-dependent effects on CD4^+^ T cell activation require expression of C15 by the APC or if C15 can block CD4^+^ T cell activation *in trans*. We transfected MHCII negative CHO cells with C15-HA, sorted cells based on HA expression, and then co-cultured them with BMDCs pulsed with peptide and antigen-specific CD4^+^ T cell hybridomas. We observed robust CD4^+^ T cell activation by BMDCs regardless of whether the co-cultured CHO cells expressed C15 (**Fig 5**), indicating that C15 must be expressed *in cis* on the APC in order to inhibit CD4^+^ T cell activation. This finding is consistent with our data in Fig 3 that C15 specifically targets direct presentation by the infected APC.

### C15 does not function by inhibiting peptide loading

**Fig 6:**
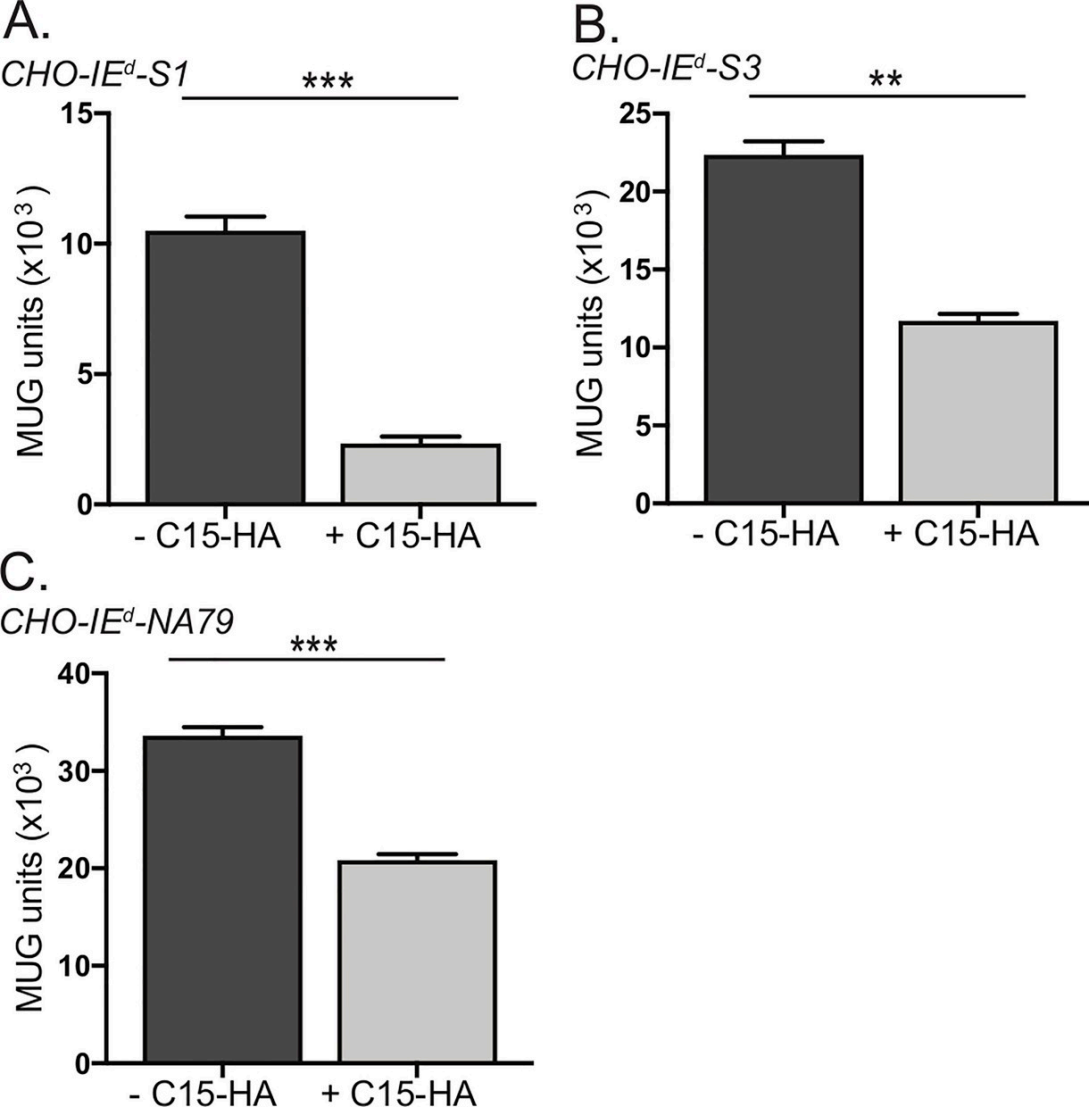
C15 does not function by inhibition of peptide loading. **A-C)** CHO cells engineered to stably express I-E^d^ covalently bound to peptide in the binding groove, either **A)** S1 **B)** S3, or **C)** NA-79 were transfected with C15-HA for 24 hours. Cells were stained for surface expression of HA and sorted based on HA expression. Cells were co-cultured with T cell hybridomas specific for each peptide. T cell activation was measured by proxy of β-galactosidase conversion of MUG substrate, with background signal from CHO parental cells cultured with T cell hybridomas subtracted from sample wells. Representative of three independent experiments. Significance analyzed by student’s T test, **p<0.01, *** p<0.001, error bars signify square root of the squared SEMs.

The previous experiments do not rule out the possibility that C15 functions by inhibiting peptide loading onto MHCII during MHCII maturation. To investigate this possibility, we analyzed whether C15 inhibition could block T cell activation by pre-loaded peptide-MHCII. We generated a series of CHO recombinant cell lines that stably expressed an MHCII (IE^d^ allele) molecule containing a peptide covalently bound within the binding groove (**S4 Fig**). We transfected three independent cell lines (each presenting a different IE^d^-restricted influenza epitope) with C15-HA, sorted populations based on HA tag expression, and subsequently co-cultured the sorted populations overnight with CD4^+^ T cell hybridomas recognizing each of the three epitopes. We observed that all three MHCII-single chain epitope constructs were significantly less able to activate their cognate CD4^+^ T cell hybridoma in the presence of C15 (**Fig 6A–6C**). This indicates that C15-mediated inhibition operates independent of peptide loading.

### C15 suppresses superantigen-mediated CD4^+^ T cell activation

**Fig 7:**
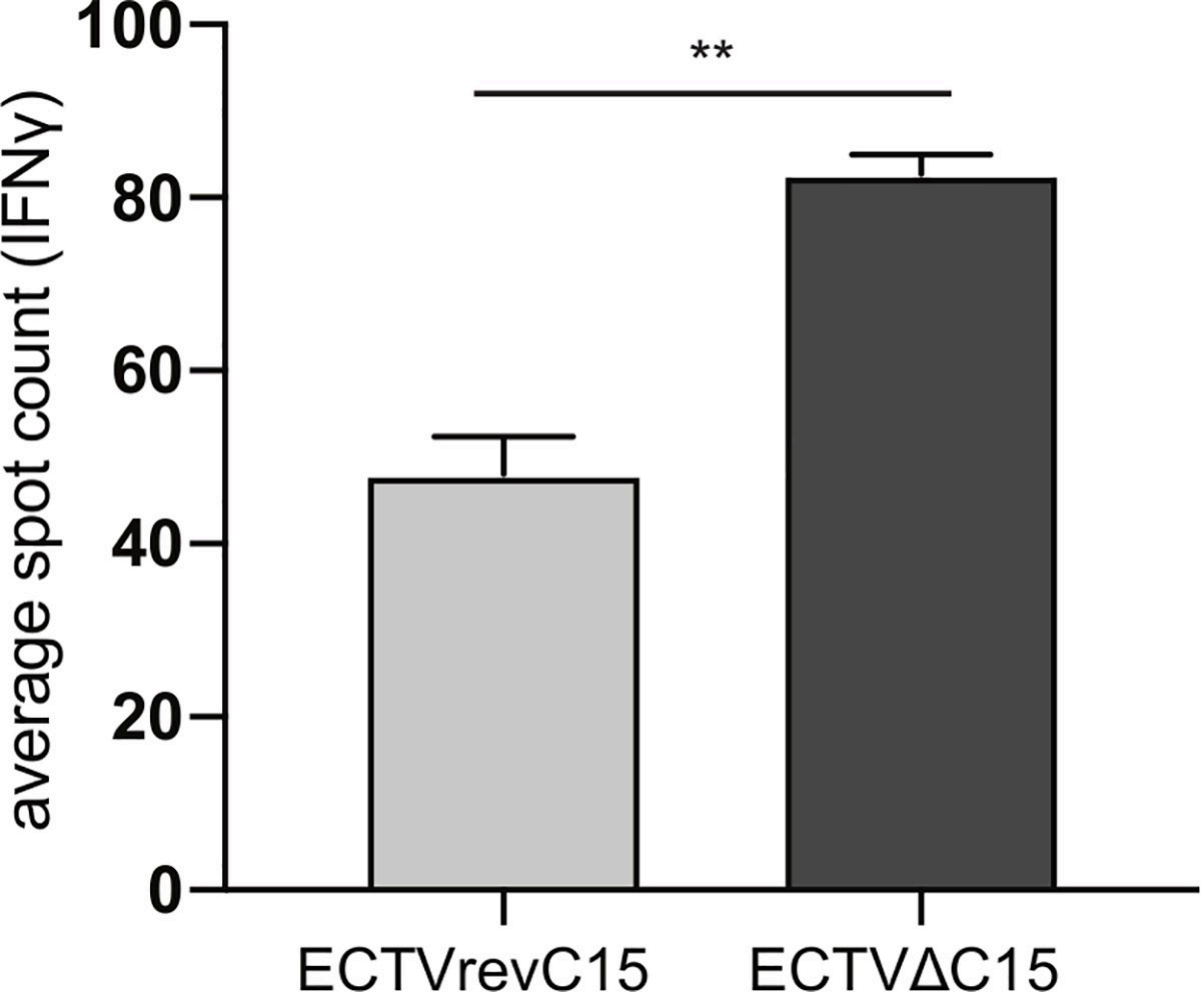
C15 prevents superantigen-mediated CD4^+^ T cell activation. PR8-specific CD4^+^ cells were co-incubated with BMDCs infected with ECTVΔC15 or ECTVrevC15 in the presence of 2 ug/mL SEB and analyzed for IFNγ production by ELISpot. Representative of three independent experiments. Significance analyzed by student’s T test, **p<0.01, error bars signify square root of the squared SEMs.

In light of C15 functioning independent of peptide loading, we asked whether C15 could also prevent superantigen- mediated T cell activation, which crosslinks MHCII and the TCR without a requirement for MHC-loaded peptide [66, 67]. We isolated splenic CD4^+^ T cells from mice infected with PR8 influenza as a source of activated T cells for analysis of superantigen-mediated stimulation. These were co-cultured with BMDCs infected with either ECTVΔC15 or ECTVrevC15 in the presence of Staphylococcal enterotoxin B (SEB) and analyzed for T cell activation via IFNγ ELISpot. SEB stimulated significantly fewer CD4^+^ T cells in the presence of C15 (**Fig 7**). The result solidifies the conclusion that the C15 mechanism of action does not entail inhibition of peptide loading.

### C15 specifically inhibits CD4^+^ T cell synapse formation

**Fig 8:**
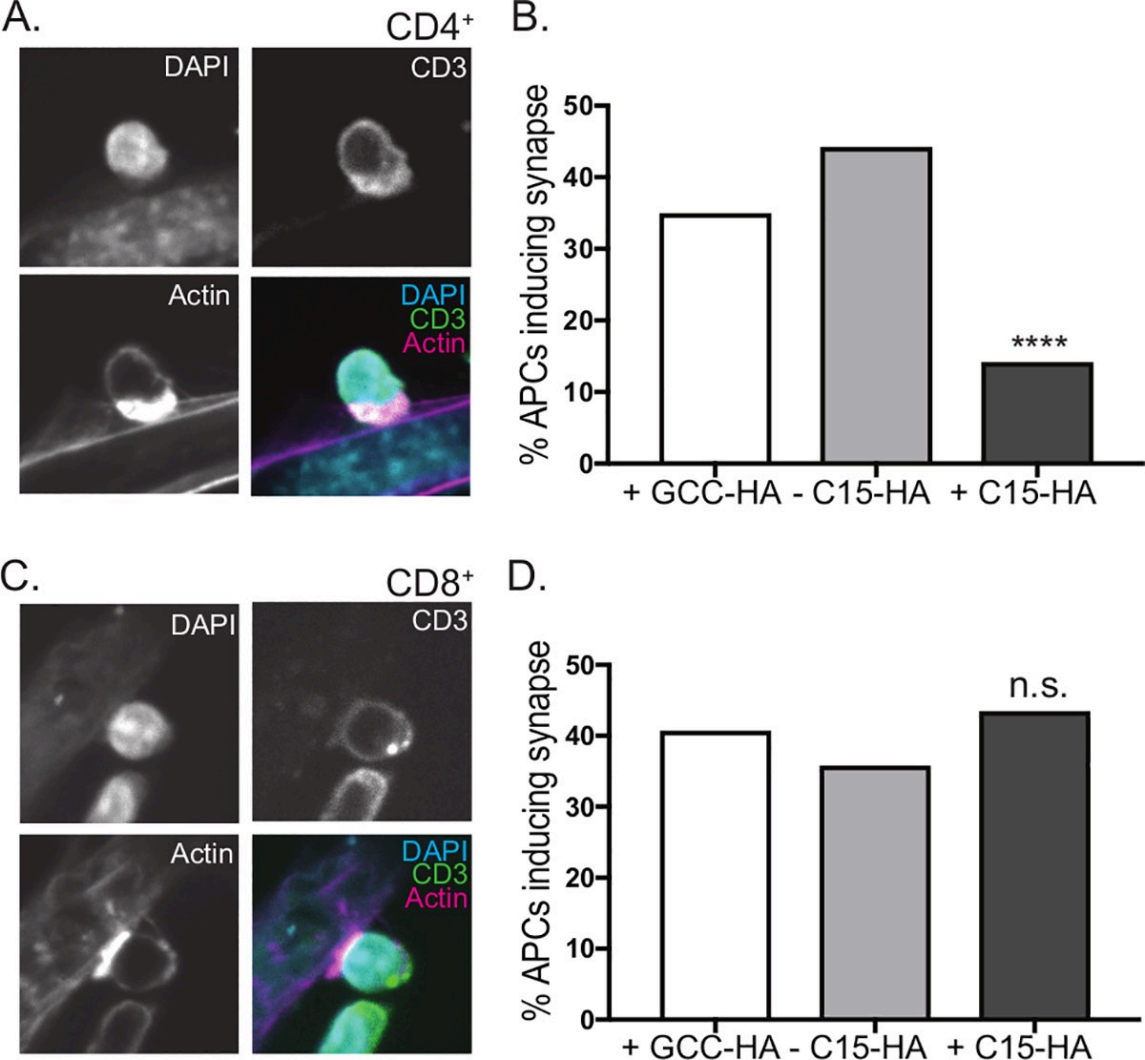
C15 specifically inhibits CD4^+^ T cell synapse formation. B6-IE^d^ fibroblasts pulsed with SIINFEKL peptide were transfected with C15-HA or GCC-HA for 24 hours. Cells were co-cultured with **A,B)** CD4^+^ T cells from a naïve TEα mouse (TCR transgenic against Eα-I-A^b^) or C,D. CD8^+^ T cells from a naïve OT-I mouse (TCR transgenic against SIINFEKL-K^b^). Following co-culture, plates were gently washed, fixed, and stained with DAPI as well as antibodies against actin, HA and CD3. **A,C)** Representative images of a **A)** CD4^+^ T cell or **C)** CD8^+^ T cell forming a synapse with untransfected B6-IE^d^ fibroblast. **B,D)** The percent of B6-IE^d^ fibroblasts expressing GCC-HA or C15-HA or C15-HA negative cells in proximity to C15-HA+ cells inducing **B)** CD4^+^ T cell or **D)** CD8^+^ T cell synapse formation. This data is the pooled cell counts from at least two independent experiments per condition. Significance analyzed by Chi Square test, ****p<0.0001.

Thus far we have demonstrated that the mechanism of C15 inhibition does not involve downregulation of MHCII, CD80 or CD86, requires C15 to be on the same cell as the MHCII, and is not attributable to interference with loading of peptide onto MHCII. In light of these findings, as well as the localization of C15 at the plasma membrane (**S3 Fig**), we investigated whether C15 expression results in blockage of CD4^+^ T cell immunological synapse formation. This line of investigation was important for mechanistic insight since T cell activation can be inhibited without disruption of the synapse [68]. Accordingly, we analyzed synapse formation via confocal microscopy in the presence and absence of C15. For these studies we utilized the B6-IE^d^ cells, which constitutively produce the Eα-IA^b^ complex and can be tested for MHCI presentation by peptide pulsing with the K^b^-restricted SIINFEKL peptide. In preliminary experiments, we confirmed that these cells can promote T cell synapse formation by co-culturing B6-IE^d^ cells with T cells from TCR transgenic mice, either CD4^+^ T cells from naïve TEα (which recognize Eα-IA^b^) [69] (**Fig 8A**) or CD8^+^ T cells from naïve OT-I (which recognize SIINFEKL-K^b^) [70] (**Fig 8C**). To analyze the impact of C15 on T cell synapse formation, we transfected B6-IE^d^ cells with either C15-HA or HA-tagged Guanyl Cyclase C- (GCC-HA), an unrelated large membrane protein, and co-cultured these APCs with either CD4^+^ T cells from TEα mice or CD8^+^ T cells from OT-I mice. We assessed T cell synapse formation by staining for F-actin, as F-actin accumulation at the APC/T cell interface in the T cell is a well characterized event in synapse formation [71] (**Fig 8A and 8C**). We counted the number of B6-IE^d^ APCs, either +GCC-HA, +C15-HA or -C15-HA (from same well as +C15-HA), that formed unambiguous synapses with T cells, either CD4^+^ (**Fig 8B**) or CD8^+^ (**Fig 8D**). We observed that CD4^+^ T cells significantly less frequently formed a synapse with a +C15-HA B6-IE^d^ cell. In contrast, CD8^+^ T cell synapse formation was unaffected by the presence of C15. Furthermore, transfection with the unrelated GCC-HA membrane protein did not affect synapse formation of either CD4^+^ or CD8^+^ T cells. From these data, we conclude that C15 expression prevents formation of a functional CD4^+^ T cell synapse.

## Discussion

Here we have demonstrated that the orthopoxvirus ECTV, the causative agent of mousepox, targets direct MHCII processing and presentation to CD4^+^ T cells. Furthermore, we show that the B22 family protein C15, a virulence factor not involved in virus replication, is necessary and sufficient to inhibit CD4^+^ but not CD8^+^ T cell activation. Importantly, C15-mediated inhibition is contingent upon expression by the APC, rather than a bystander cell. Furthermore, we provide evidence that C15 does not function by downregulating surface MHCII or co-activation proteins or by blocking peptide loading. Rather, C15 functions post-peptide loading, resulting in substantially diminished CD4^+^ T cell synapse formation, the crucial early step in the CD4^+^ T cell activation program. The only other viral protein we are aware of that functions independently of MHCII surface downregulation or peptide loading is gp42 of EBV, which sterically hinders the interaction between human TCRs and MHCII [15, 16] by binding the β1 domain of MHCII, near the peptide binding groove [16]. Notably, this protein is soluble, implying a *trans* effect and a fundamentally different mechanism. Furthermore, as EBV is a persistent virus that specifically replicates in B cells and ECTV is an acute virus with broad cell tropism, these viruses have likely evolved to inhibit CD4^+^ T cells for distinct purposes.

Both ECTV and C15 specifically have been previously implicated in interference with CD4^+^ T cell function, however without elucidation of underlying mechanism. ECTV has been suggested to interfere with dendritic cell activation, though the responsible viral factor was not identified [72, 73]. A previous publication described C15 as a virulence factor that targets both CD4^+^ and CD8^+^ T cells [56]. However, mechanism of action was not explored and outcomes did not allow for distinction between direct inhibition of both T cell subsets and the possibility of indirect effects on CD8^+^ T cells via CD4^+^ T cell repression. Here we directly examined the effect of C15 on CD4^+^ and CD8^+^ T cell activation both in the context of viral infection and expression of C15 in isolation. In a variety of experimental settings, with both hybridomas and primary *ex vivo* cells, we did not observe inhibition of CD8^+^ T cells. While we measured only IFNγ to assess primary CD8^+^ T cell activation, our observation that MHCI synapse formation was unimpacted by C15 expression implies that we did not overlook inhibition of alternative CD8^+^ T cell effector functions.

Several members of the B22 family have been implicated as critical poxviral virulence factors [56, 60, 74, 75] but interestingly, various lines of evidence suggest that different members of the B22 family have distinct mechanisms of action despite relatively high conservation at the sequence level. The Früh lab has demonstrated that the MPXV B22 family protein, MPXV197, is capable of inhibiting both CD4^+^ and CD8^+^ T cells [60], in contrast to our work with C15. Furthermore, C15 requires synthesis in an MHCII^+^ APC in order to inhibit T cell activation, whereas MPXV197 can function *in trans* when present on a bystander non-APC. These data suggest intriguing possibilities for structural differences between C15 and MPXV197 that dictate the respective functions of the two proteins. For example, it is currently unknown whether the N terminus of C15 is intracellular; the N-terminus of the MPXV homolog was demonstrated to be intracellular [60] while the orientation of the CPXV homolog could not be resolved [63]. Greater understanding of the specific inhibitory mechanisms employed will likely require more detailed structural information on the various B22 family members.

That C15 localizes to the plasma membrane of the APC and hinders superantigen-mediated CD4^+^ T cell activation offers intriguing mechanistic possibilities. As SEB is known to interact with the α-chain of MHCII near but outside of the peptide binding groove [76], it is possible that C15 sterically hinders the interaction of MHCII with the TCR or SEB without altering the conformation of MHCII. Likewise, C15 might force structural alterations in MHCII that decrease affinity for TCR interaction. An intriguing alternative is that C15 interferes with MHCII movement within the plasma membrane, possibly sequestering MHCII outside of activating lipid rafts, which are critical for both SEB-mediated T cell activation and productive immunological synapse generation [77]. Ongoing studies are exploring these possibilities and whether the mechanism requires direct interaction between C15 and MHCII. In addition, the selective impact of C15 on CD4^+^ but not CD8^+^ T cell synapse formation could be a useful tool for greater understanding of the mechanisms underlying activation of these two subsets. Indeed, while the synapse structure is known to differ depending on cytolytic capacity of the T cell [78–80], there are differences between CD4^+^ and CD8^+^ T cell synapses independent of this property [81].

The critical first step in CD4^+^ T cell activation comes from engagement of the T cell receptor with a foreign peptide being presented in complex with MHCII molecules. Historically, these peptides were thought to originate from material endocytosed by a professional APC for subsequent degradation and loading onto immature MHCII molecules within the endocytic network [1]. However, there is increasing evidence that endogenous MHCII antigen processing and presentation by a directly infected cell can drive CD4^+^ T cell activation [17–24] and, in the case of influenza, accounts for the large majority of CD4^+^ T cell expansion [24]. Importantly, many viruses are able to infect professional APCs such as dendritic cells and most cells can upregulate MHCII when stimulated with IFNγ [82, 83], suggesting that direct endogenous MHCII processing is a common phenomenon during viral infection. If endogenous MHCII presentation by infected cells is an important aspect of host defense, then pathogens can be expected to develop strategies to overcome this system. Indeed, most of the reported viral mechanisms for inhibition of MHCII processing and presentation depend on direct infection of the APC, for example, repression of MHCII transcription and forced degradation of mature MHCII molecules [2]. During ECTV infection it is known that infected MHCII^+^ cells facilitate viral systemic spread [84, 85] and that a cytolytic CD4^+^ T cell response is elicited [54]. The increased viral virulence in the presence of C15 suggests that direct presentation might also be critical in identifying virally infected cells for targeting by cytolytic CD4^+^ T cells.

Previous studies from our lab have demonstrated robust epitope-specific CD4^+^ T cell activation during ECTV infection [57–59], suggesting that blockade of direct endogenous MHCII presentation is bypassed in some way. While C15 inhibition is robust, it is not complete, and this may provide a partial explanation. More intriguing is the possibility that indirect MHCII presentation is a major driver of ECTV-specific CD4^+^ T cell responses *in vivo*. This would contrast with the minor role played by indirect presentation in influenza infections [24]. Our *in vitro* results support this hypothesis and future *in vivo* studies will seek to test this directly. Currently, little is known about the cellular machinery that supports indirect presentation and the extent to which classical exogenous antigen processing is used for this purpose. Furthermore, it is possible that the necessity of relying on indirect processing for MHCII presentation results in less robust CD4^+^ T cell responses due to decreased diversity of peptide display. That ECTV appears to utilize indirect MHCII presentation, perhaps due to C15 evolutionary pressure, suggests that this system could be a useful model for greater understanding of alternative MHCII presentation pathways.

In this work we demonstrate that ECTV employs a novel mechanism for evading CD4^+^ T cell responses which results in inhibition of MHCII/TCR synapse formation. This adds to the relatively limited list of pathogen-encoded inhibitory strategies that target CD4^+^ T cell activation [2]. While much of the literature to date has focused on viral inhibition of MHCI processing and presentation, with increased interest in CD4^+^ T cells and the elucidation of a more complex MHCII processing and presentation landscape, it seems likely that many more instances of viral targeting of the MHCII processing and presentation system remain to be identified.

## Materials and methods

### Ethics statement

Animal studies performed in this study fall under an animal protocol approved by the Children’s Hospital of Philadelphia Research Institute Institutional Animal Care and use Committee (protocol id 15–001314). The Children’s Hospital of Philadelphia is an AAALAC accredited institution and adheres to the standards set by the Animal Welfare Act and the NIH Guide for the Care and Use of Laboratory Animals.

### Primary APCs and cell lines

Bone marrow dendritic cells (BMDCs) were derived from the bone marrow of pooled female C57BL/6 mice (The Jackson Laboratory, 00064) and cultured for 7–10 days in RPMI 1640 (10% FBS, antibiotics, L-glutamine, and 2-ME) with GM-CSF (Gemini Bio-Products). B6-IE^d^ fibroblasts have been previously described [22, 24, 65] and were cultured in DMEM supplemented with 5% FBS, antibiotics and L-glutamine. L929 cells (ATCC CCL-1) were cultured in DMEM supplemented with 5% FBS, antibiotics and L-glutamine. BSC-1 cells were cultured in DMEM supplemented with 10% FBS, antibiotics and L-glutamine. T cell hybridomas previously described [21, 24, 86, 87] were maintained in RPMI supplemented with 10% FBS, 1×penicillin/streptomycin, 1×L-glutamine and 0.05 mM 2-ME. The epitope-linked MHCII stable CHO cell lines, similar to previous reports [88], were generated via double transfection of pCMV-IE^d^-α and pCMV-IE^d^-β-epitope plasmids. The epitopes (S1, S3, and NA79) were linked to IE^d^-β via an N-terminal poly-4xGly-Ser linker. Transfection was accomplished with Lipofectamine 2000 (ThermoFisher 11668) reagent in 24-well plates following the manufacturers recommended protocol. After 48 hours, the transfected cells were placed under Geneticin (500ug/mL) selection for 5 days. After selection, IE^d^ expression was verified by flow cytometry (clone M5.114.15.2, Biolegend). Epitope expression was verified via MUG assay by co-incubating the CHO-IE^d^-epitope stable cell lines with T-cell hybridomas specific to S1, S3, and NA79 (**S4F Fig**). CHO-IE^d^-epitope cell lines were cultured in DMEM supplemented with 5% FBS, antibiotics and L-glutamine.

### Mouse models of infection

C57Bl/6 (JAX 000664) and BALB/c (JAX 000651) mice were purchased from Jackson Laboratories. C57Bl/6 mice were infected with either 3 x 10^3^ pfu of ECTV or alternatives as described below. Spleens from naïve TEα mice were a kind gift from the lab of Ivan Maillard (University of Pennsylvania). Spleens from naïve OT-I mice were a kind gift from the Christopher Hunter (University of Pennsylvania) and Edward Behrens labs (Children’s Hospital of Philadelphia Research Institute). All mice utilized for this study were females ranging from 6–10 weeks in age. All animals were age matched within an individual experiment.

### Viruses and virus purification

The following poxviruses viruses were used during the course of this work: ECTV expressing GFP (Moscow strain background; gift of Dr. Luis Sigal [62]), ECTVΔC15 (Moscow strain), and ECTVrevC15. ECTVΔC15 was constructed using standard homologous recombination techniques [56]. Approximately 75% of the C15 open reading frame was replaced with the coding sequence for GFP under the transcriptional control of the poxvirus p7.5 early/late promoter. After the infection/transfection stage, a GFP-positive plaque was isolated and subsequently underwent five rounds of passage in BS-C-1 cells to ensure that 100% of plaques stably expressed GFP. As a control, we also constructed a revertant virus (ECTVrevC15) in which the native open reading frame was recombined back into ECTVΔC15. For virus purification, TK^-^ cells were infected for 72 hours followed by lysis using repeated freeze, thaw, sonication cycles. The cell lysate was then purified through a 36% sucrose cushion and resuspended in 1 mM Tris-HCL for use in animal and *in vitro* experiments. Viral titers were obtained via plaque assay on BSC-I cells. Influenza A virus, A/Puerto Rico/8/1934 (PR8), subtype H1N1 was grown, harvested, titered, and plaqued from isolates as described previously [21, 24, 89]. One hemagglutinating unit (HAU) of live PR8 virus ≈1.5 × 10^5^ plaque-forming units, titered as previously described [89].

### C15-HA plasmid generation

A C15 ORF codon optimized for human cells was generated commercially (GenScript). Using standard PCR and restriction enzyme-based cloning, the ORF was inserted into the commercially available CMV c-terminal- HA tag plasmid (Clontech 635690) via *Sal*1 and *Kpn*1 restriction enzyme sites. Ampicillin-resistant colonies were screened using restriction enzyme digestion following plasmid isolation. Insert expression was verified by a Western blot against the HA tag after transfection into 293T cells.

### ELISpot assay

Female C57BL/6 mice 6–8 weeks of age were inoculated with appropriate virus (three mice per group). ELISpot plates were coated with anti–IFNγ (BD Biosciences ELISpot mouse IFNγ Ab pair) at 1:200 in PBS and incubated overnight at 4°C. Ten days post-infection, spleens from each group were harvested. CD4^+^ or CD8^+^ T cells were purified from bulk splenocytes using negative bead isolation (Invitrogen Dynabeads Untouched Mouse CD4^+^ or CD8^+^ Isolation Kit) and incubated with the indicated APCs, with three technical replicates per condition. After an overnight incubation, the plates were developed (BD Biosciences ELISpot mouse IFNγ Ab pair and AEC Substrate Kit), and IFNγ spots produced by activated T cells were counted (ImmunoSpot reader); background signal from mock infection was subtracted from sample wells.

### In vitro infections for ELISpot and flow cytometry

BMDCs or L929 cells were infected in low volume 0.1% BSA/PBS at a multiplicity of infection (MOI) of 1 for 1.5 hours 37°C with agitation followed by addition of RPMI 1640 (10% FBS, antibiotics, L-glutamine, and 2-ME). Cells were then incubated at 37°C overnight, with an infection rate of 50–60% observed by flow cytometry. Following infection, cells were washed and re-suspended for ELISpot or flow staining. For ELISpot, prior to co-culture of infected cells with T cells, neutralizing antibodies were added to prevent viral spread (anti-B5R and anti-L1R). The following reagents were obtained through BEI Resources, NIAID, NIH: Monoclonal Anti-Vaccinia Virus (WR) B5R Protein, Residues 20 to 275 (Ectodomain), (similar to VMC-20), produced *in vitro*, (NR-50418); Vaccinia Virus (WR) L1R Protein with C-Terminal Histidine Tag, Recombinant from Baculovirus, (NR-21986).

### Focus forming assay

BSC-1 cells were seeded to confluency in 96-well flat bottom dishes overnight. The following day, infection samples were serially diluted in triplicate, added to wells (having removed culture media prior) in 50 uL volume and incubated at 37°C for 1 hour. Samples were removed from wells and avicel overlay (1.25% avicel (FMC RC-591 NF) in DMEM) added to wells. The plates were incubated for 18 hours, prior to fixation with 4% PFA, permeabilization with 0.5% Triton-X in PBS, and blocking with 5% BSA in TBS-T. Wells were stained with Rb anti-VACV Ab (Thermo PA1-7258) followed by HRP Gt anti-Rb secondary (CST 7074S). Lastly wells were incubated with KPL TrueBlue TMB substrate (SeraCare 5510–0049) for 2 hours, washed with water and air dried. Positively-stained virus-infected foci were counted (ImmunoSpot Reader).

### In vivo viral virulence assay

Female BALB/c mice were inoculated with 3 x 10^2^ pfu ECTVΔC15 or ECTVrevC15 in the footpad. Mice were weighed on alternate days and monitored for signs of foot pathology as well as morbidity and mortality daily. Mice that displayed persistent recumbency or lost over 20% of initial body weight were sacrificed in accordance with the Institutional Animal Care and Use guidelines of the Children’s Hospital of Philadelphia IACUC.

### Transfections

Cells were plated in 10cm tissue culture dishes in most cases or in chambered coverglass slides (Thermofisher) (**Fig 8**) overnight. At 70–80% confluency cells were transfected with 15 ug or 1ug (**Fig 8**) of the indicated plasmid using XtremeGENE HP reagent (Sigma Aldrich) in OPTI-MEM (Thermofisher) in DMEM supplemented with 5% FBS and L-glutamine. At 24 hours post transfection cells were harvested for sorting or prepared for synapse formation assay.

### Flow cytometry

Cells were stained with live/dead Aqua (Thermofisher L34965) prior to staining with anti-MHCII (clone M5.114.15.2, Biolegend), anti-CD80 (clone 16-10A1, Biolegend) and anti- CD86 (clone GL-1, Biolegend) (**Fig 4**) on ice for 30 minutes. Cells were washed and fixed in 2% para-formaldehyde (VWR 76221–378) prior to acquisition on an LSRII instrument. For sorting experiments cells were stained with live/dead Aqua (Thermofisher L34965) prior to staining with anti-HA (clone 16B12, Biolegend) and sorted using a MoFLo Astrios or FACS Jazz instrument (Children’s Hospital of Philadelphia Research Institute Flow Cytometry Core Facility).

### In vitro antigen-presentation (MUG) assay

Following transfection and sorting, the indicated cells were co-cultured with epitope-specific *LacZ*-inducible T cell hybridomas overnight in 384 well plates (Sigma CLS3571). In indicated cases, 0.2 mg/mL exogenous peptide was added (>85% purity, Genscript, resuspended in DMSO). Activation was measured by detection of fluorometric β-galactosidase substrate methyl-umbelliferyl-β-D-galactopyranoside (MUG; Sigma M1633) as previously described [21, 24, 86]. With the exception of SF.4, the background signal from mock peptide (DMSO) or other appropriate negative control denoted in the figure legend was subtracted from the sample signal. Fluorescence was detected on Tecan Infinite M200 Pro Plate Reader.

### Superantigen activation ELISpot

The ELISpot as described above was modified in the following manner. Splenic CD4^+^ T cells were isolated from C57Bl/6 mice infected intraperitoneally for 10 days with 4 HAU of PR8 influenza as previously described [24]. At the time of BMDC/T cell co-culture in the ELISpot plate, 2 ug/mL recombinant SEB (Toxin Technologies Inc.) was added to the appropriate wells, with three technical replicates per condition. Background signal from mock treated wells was subtracted from sample wells.

### Synapse formation assay

B6-IE^d^ fibroblasts were plated on Lab-Tek II chambered coverglass slides (Thermo Fisher 155409) and allowed to attach overnight. The cells were then transfected with either C15-HA or Guanyl Cyclase C-HA (GCC-HA) as a control, using XtremeGENE HP reagent (Sigma Aldrich). After 24 hours, CD4^+^ T cells from TEα mice or CD8^+^ T cells from OT-I mice were allowed to settle on the APCs in L15 media (Gibco) supplemented with 2mg/mL glucose. After a 30 minute incubation to allow synapse formation, co-cultures were gently washed and fixed with 3.7% PFA (Electron Microscopy Science) for 15 mins, then blocked and permeabilized in PBS, 0.01% saponin, 0.05% fish skin gelatin (PSG) for 20 min. Cells were stained with anti-HA (clone 16B12, Biolegend), anti-CD3 (clone 17A2, Biolegend), fluorescent phalloidin (Life Technologies Molecular Probes), and Hoechst (Life Technologies Molecular Probes) for 1 hour in PSG. Cells were then washed 3x with PSG, and stained with the appropriately conjugated secondary antibodies (Life Technologies Molecular Probes) for 1 hour in PSG. Cells were washed 3x with PSG, mounted, and imaged using a 63x PlanApo 1.4 NA objective on an Axiovert 200M (Zeiss) with a spinning disk confocal system (Ultraview ERS6; PerkinElmer). For quantification, C15-HA or GCC-HA expressing cells were located, and scored whether for the presence or absence of a T cell forming a synapse with >50 cells counted per condition. HA-negative surrounding cells were also scored. To capture representative images, multiple z-planes spanning a total of 10μm were collected with an Orca Flash 4.0 camera (Hamamatsu) and prepared using ImageJ software; an individual plane is pictured.

## Supporting information

S1 Fig ECTVΔC15 retains virulence in the absence of C15. BALB/c mice were infected with ECTVΔC15 and monitored daily for foot loss. The death of mice prior to potential foot loss precluded similar analysis with ECTVrevC15. Representative of two independent experiments.
(TIF)

S2 Fig C15 restricts endogenous but not indirect MHCII presentation. Three female C57Bl/6 mice were infected with 3x10^3^ pfu WT ECTV via footpad injection. Ten days later, mice were sacrificed and spleens were pooled. CD4^+^ T cells were isolated by negative bead selection and in the presence of neutralizing antibodies mixed with either BMDCs infected with ECTVΔC15 or ECTVrevC15 (direct presentation) or infected fibroblasts and uninfected BMDCs (indirect presentation). CD4^+^ T cell activation was measured via IFNγ production by ELISpot. Representative of 3 independent experiments. Significance analyzed by student’s T test, ***p<0.001, error bars signify square root of the squared SEMs.
(TIF)

S3 Fig The C terminus of C15 is extracellular. B6-IE^d^ fibroblasts were transfected with C15-HA prior to staining for surface expression of HA tag.
(TIF)

S4 Fig CHO-IE^d^-epitope stable cell lines activate T cell hybridomas in an epitope specific manner. Epitope expression in these stable cell lines was verified by co-culturing these cells with T cell hybridomas specific for each peptide. T cell activation was measured by proxy of β-galactosidase conversion of MUG substrate.
(TIF)

S1 Data Excel spreadsheet containing, in separate sheets, the underlying numerical data and statistical analysis for Figure panels 1a, 1b, 2b, 2c, 2d, 3a, 3b, 3c, 3d, 4a-f, 6a, 6b, 6c, 7, 8b, 8d, SF1, SF2, SF4. (XLSX)
