# Supplementary figures and images for: Ectromelia-encoded virulence factor C15 specifically inhibits antigen presentation to CD4+ T cells post peptide loading
Source: PLoS Pathog. 2020 Aug 3;16(8):e1008685. doi: 10.1371/journal.ppat.1008685 (PMC7425992; doi:10.1371/journal.ppat.1008685)

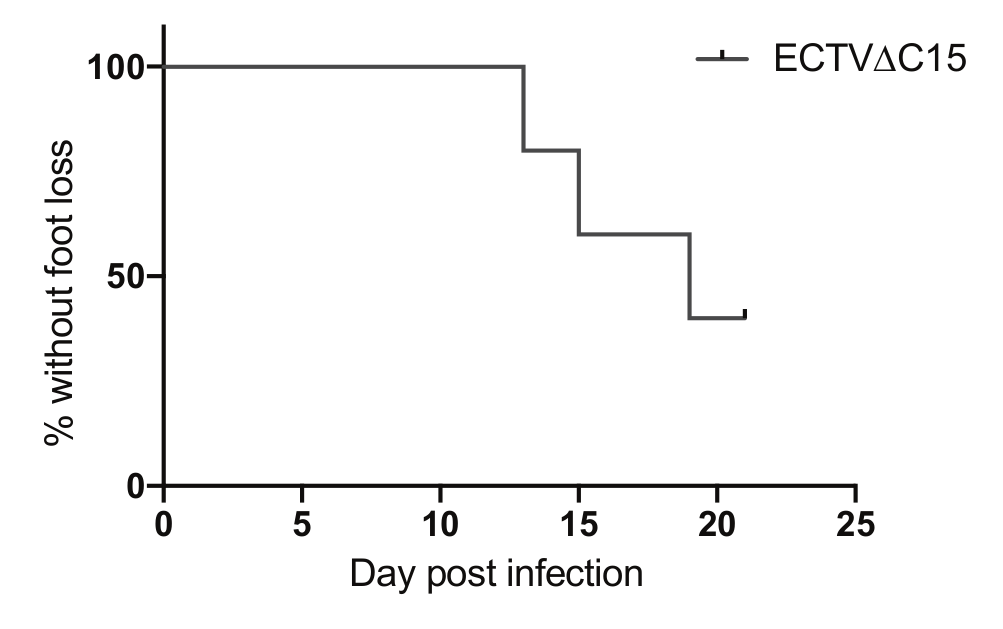

Supplement: S1 Fig — BALB/c mice were infected with ECTVΔC15 and monitored daily for foot loss. The death of mice prior to potential foot loss precluded similar analysis with ECTVrevC15. Representative of two independent experiments. (TIF) [file ppat.1008685.s001.tif]

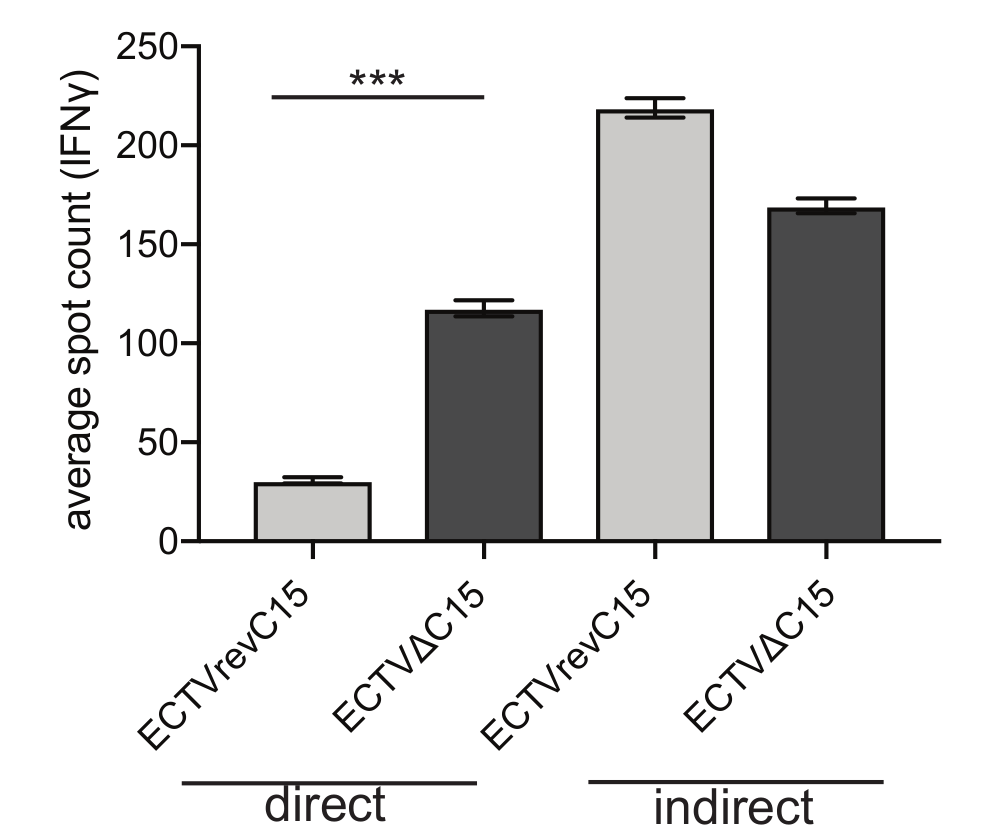

Supplement: S2 Fig — Three female C57Bl/6 mice were infected with 3x10^3^ pfu WT ECTV via footpad injection. Ten days later, mice were sacrificed and spleens were pooled. CD4+ T cells were isolated by negative bead selection and in the presence of neutralizing antibodies mixed with either BMDCs infected with ECTVΔC15 or ECTVrevC15 (direct presentation) or infected fibroblasts and uninfected BMDCs (indirect presentation). CD4+ T cell activation was measured via IFNγ production by ELISpot. Representative of 3 independent experiments. Significance analyzed by student’s T test, ***p<0.001, error bars signify square root of the squared SEMs. (TIF) [file ppat.1008685.s002.tif]

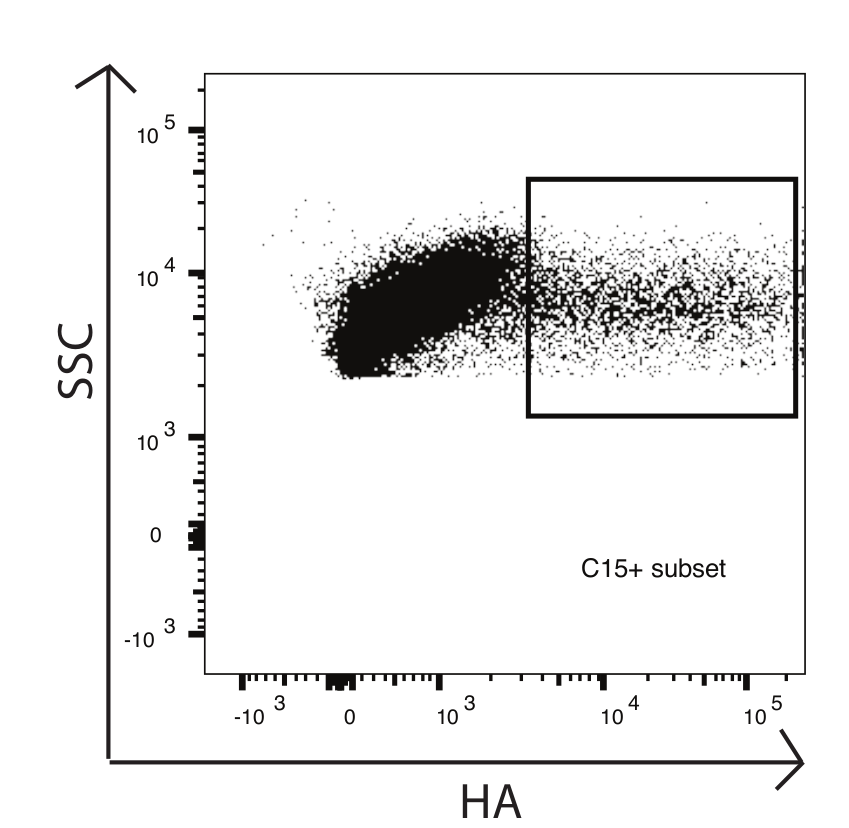

Supplement: S3 Fig — B6-IEd fibroblasts were transfected with C15-HA prior to staining for surface expression of HA tag. (TIF) [file ppat.1008685.s003.tif]

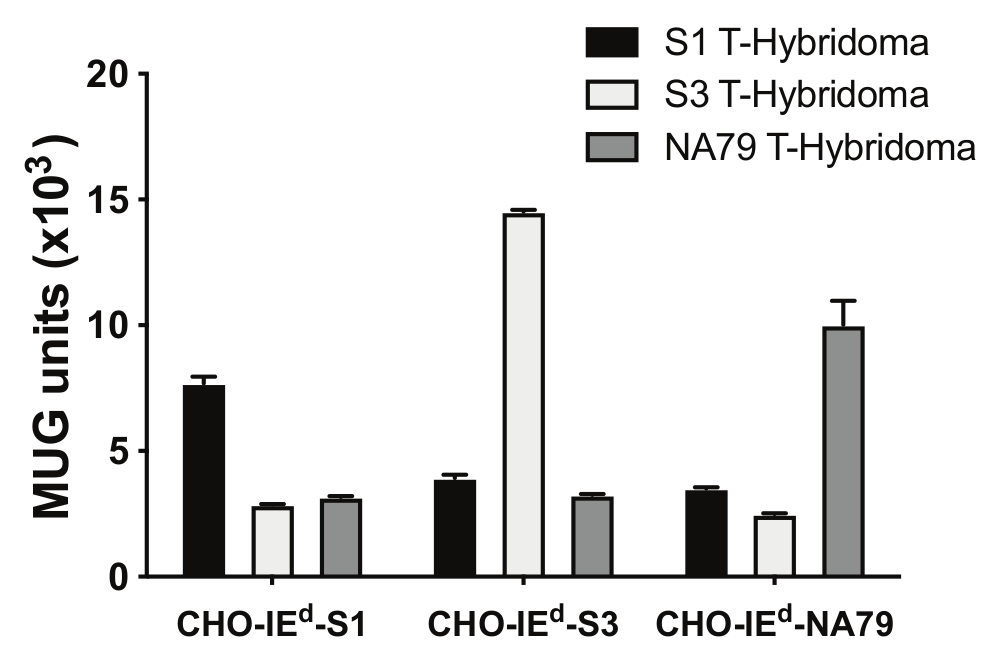

Supplement: S4 Fig — Epitope expression in these stable cell lines was verified by co-culturing these cells with T cell hybridomas specific for each peptide. T cell activation was measured by proxy of β-galactosidase conversion of MUG substrate. (TIF) [file ppat.1008685.s004.tif]
